# Using Visual Timelines in Telephone Interviews: Reflections and Lessons Learned From the Star Family Study

**DOI:** 10.1177/1609406920913675

**Published:** 2020-03-30

**Authors:** Bethan Pell, Denitza Williams, Rhiannon Phillips, Julia Sanders, Adrian Edwards, Ernest Choy, Aimee Grant

**Affiliations:** 1 Centre for Trials Research, College of Biomedical & Life Sciences, Cardiff University, Cardiff, United Kingdom; 2 Division of Population Medicine, School of Medicine, Cardiff University, Cardiff, United Kingdom; 3 Cardiff School of Sport & Health Science, Cardiff Metropolitan University, Cardiff, United Kingdom; 4 School of Healthcare Sciences, Cardiff University, Cardiff, United Kingdom; 5 Division of Infection and Immunity, School of Medicine, Cardiff University, Cardiff, United Kingdom; 6 Independent Researcher, Cardiff, South Glamorgan, United Kingdom

**Keywords:** communication, data collection, life stories, lived experience, power, empowerment, interviews, marginalized or vulnerable populations, reflexivity, research participation, qualitative methods, visual methods

## Abstract

Visual timeline methods have been used as part of face-to-face qualitative interviewing with vulnerable populations to uncover the intricacies of lived experiences, but little is known about whether visual timelines can be effectively used in telephone interviews. In this article, we reflect on the process of using visual timelines in 16 telephone interviews with women as part of the “STarting a family when you have an Autoimmune Rheumatic disease” study (STAR Family Study). The visual timeline method was used to empower women to organize and share their narratives about the sensitive and complex topic of starting a family. We conducted a thematic analysis of the audio-recorded interview data, using researchers’ field notes and reflections to provide context for our understanding of the benefits of using timelines and to understand the process of using visual timelines during telephone interviews. Resource packs were sent to women before study participation; 11 of the 16 women completed a version of the timeline activity. Six themes were identified in the methodological data analysis: (1) use and adaptation of the timeline tool, (2) timeline exchange, (3) framing the interview: emphasizing that women are in control, (4) jumping straight in, (5) taking a lead, and (6) disclosing personal and sensitive experiences. The use of visual timelines facilitated interviewee control and elicited rich narratives of participants’ experiences in telephone interviews. Women created their visual timelines autonomously and retained ownership of their timeline data; these features of the data generation process need to be considered when using visual timelines in telephone rather than face-to-face interviews. Use of visual methods within telephone interviews is feasible, can generate rich data, and should be further explored in a wider range of settings.

## Introduction

### Background to the STAR Family Study

The “STarting a family when you have an Autoimmune Rheumatic disease” (STAR Family Study) adopted a mixed-methods approach to help construct a holistic picture of women’s transitions to motherhood when they had an autoimmune rheumatic disease (ARD, Phillips, Pell et al., 2018; Phillips, Williams et al., 2018). ARDs are debilitating and painful long-term conditions where the immune system attacks its own tissues such as inflammatory arthritis, systemic lupus erythematosus, and vasculitis (Goldblatt & O’Neil, 2013). Women with ARDs encounter a range of challenges when they begin to approach a transitional journey during their childbearing years, including fertility, timing pregnancies, increased risk of miscarriage, changes in disease activity, risks and benefits of medication and treatment during pregnancy and breastfeeding, and managing pain and physical limitations when caring for young children (Nightingale et al., 2006; Ostensen & Ceti, 2015; Signore et al., 2011). Research has highlighted the lack of information available to women during this emotive and challenging time, and more integrated care has been recommended (Ackerman et al., 2016; Phillips, Williams et al., 2018).

Therefore, the topic we wished to explore in the STAR Family Study was of a sensitive and complex nature, with a vulnerable group of women. We adopted a woman-centered approach to address the power imbalance between the participants and researchers, as we wanted to place the women in control of sharing their narratives as experts in their own experiences, highlighting and reinforcing women’s autonomy. For the purposes of this article, we conceptualized power as something one possesses as we focused on locally situated power dynamics between the interviewee and interviewer within the context of the research interview. However, we acknowledge there are other definitions of power (e.g., Sarup, 1993). Our study ethos was consistent with feminist approaches to qualitative research (e.g., Brooks & Hesse-Biber, 2007; Hesse-Biber, 2011; Landman, 2006; Tong & Fernandes Botts, 2017) but also has relevance to other marginalized groups, aiming to achieve a better understanding of participants’ lived experiences that might otherwise be invisible or only partly observed.

In line with our study ethos, we wanted to use visual methods within this study, as graphic elicitation is a valuable method to uncover the intricacies of lived experience that might not come to light through discussion alone, particularly when exploring sensitive issues (Aarsand & Aarsand, 2018; Bravington & King, 2018; Cornwall, 1992; Gauntlett, 2007; Rose, 2007). Qualitative studies have used an array of visual methods for more creative interviewing methods including photographs (Frith & Harcourt, 2007; Heng, 2017; Radley & Taylor, 2003; Rose, 2007, 2012), Lego (Gauntlett, 2007), paintings and artwork (Irving, 2007), possessions in the home (Grant et al., 2017; Miller, 2008), and sandboxing (Mannay et al., 2017). However, methodological reflection around the appropriateness of visual tools in different contexts is essential (Mannay, 2016).

### Visual Timeline Methods

Timeline methods involve forming a visual chronological representation of significant life events (Berends, 2011; Patterson et al., 2012). Participants are able to organize their thoughts and share their lived experiences in their own way by reflecting on their past, present, and future (Bagnoli, 2009), enabling rich and unique explorations of data (Mannay, 2010, 2016). Timelines are particularly useful in illustrating narratives of individuals’ journeys (Sheridan et al., 2011) and capturing the meaning and context attached to specific events (Leung, 2010), which was an important focus within the STAR Family Study.

Previous studies have used timelines as a method of qualitative data collection to address some of the challenges arising in more traditional qualitative interviews. These include altering traditional power imbalances between the interviewer and interviewee, adopting a more person-centered approach to interviewing, facilitating interactivity to enhance the understanding of experiences, and negotiating potential barriers associated with interviewing vulnerable or marginalized groups (Berends, 2011; Goldenberg et al., 2016; Harris & Rhodes, 2018; Kolar et al., 2015).

In the STAR Family Study, it was anticipated that the women’s journeys would be evocative and poignant, causing potential distress when sharing their personal stories, which can generate ethical and practical challenges for the researcher–participant relationship (Hollway & Jefferson, 2013). Creating a timeline has been reported as cathartic; aiding reflection on positive and negative personal experiences by acting as a visual guide, framing participants’ journeys, and highlighting participants’ resilience during transitions between significant life events (Kolar et al., 2015).

We chose to use timeline-facilitated interviews in the study, over other available visual methods, as this would enable women to visualize their journey chronologically, helping them to organize their narratives and reflect on important life events along their journey so far. This would ultimately facilitate an exploration and conceptualization of the women’s personal experience and perspective.

### Use of Visual Timelines in Telephone Interviews

Our population of interest in the STAR Family Study were a hard-to-reach group, who were widely geographically dispersed across the UK. Consequently, some of the interviews needed to take place over the telephone to fit within the study’s budget and staff resources.

Telephone interviewing is becoming recognized in its own right as having the capacity to produce rich and high-quality data (Grant, 2011; Irvine, 2011; Vogl, 2013; Ward et al., 2015), with tools and strategies being developed to help qualitative researchers use them appropriately (Farooq & De Villers, 2017). The literature on visual methods timelines has focused on their use within face-to-face interviews as a coproduction method, where the participant and interviewer can interact with the visual timeline to explore the complexities of participants’ experiences (Gauntlett, 2007). However, there have been arguments around the coproductive nature of the timeline method and the potential shifts in power imbalance, as well as the possibility of participants’ feelings of oversharing experiences that they may, in hindsight, regret disclosing (Adriansen, 2012), whereas there would be limited opportunity for coproduction within telephone interviews.

The aim of this study was to assess the feasibility of using visual timelines in telephone interviews as part of the STAR Family Study, reflecting on the process of data generation and the quality of data produced when using this method.

### Research Questions

We set out to answer the following research questions:

1. How were the visual timelines used by women and researchers in the telephone interviews (e.g., what visual form did they take, who was involved in generating the timelines, were the timelines shared with the researcher and if so when)?
2. What impact did their use have on the generation of data in terms of the interviewee-interviewer dynamic and formation and sharing of women’s narratives?
3. What impact did visual timelines have on the quality of data produced in telephone interviews in terms of narrative length, detail, and coverage of sensitive and emotive topics?

## Method

The STAR Family Study used timelines as a methodological tool to facilitate face-to-face and telephone interviews with women who had an ARD and were thinking about starting a family, were currently pregnant, or were a parent to young children. We reflect on the experience of using timelines over the telephone through analysis of reflective field notes, audio recordings, and interview transcripts. This article will focus on the interactions and behavior of interviewers and participants in order to highlight methodological lessons through keys areas of study context and ethos.

### Participants

Our interview sample was derived from women who had taken part in the online survey aspect of the STAR Family Study, which had been advertised on social media, and who had reported willingness to participate in an additional in-depth interview about their experiences (Phillips, Pell et al., 2018). The women who took part were UK residents, aged 18–49 years, with a diagnosed ARD, who were thinking about starting a family, currently pregnant, or had young children. Women were purposively sampled based on their family situation, aiming for an equal representation of women at different stages of their journey (i.e., preconception, pregnant, or already had young children). Data collection took place between December 2016 and April 2017.

### Interview Preparation and Study Ethos

Authors 1 and 2 facilitated the interviews, both of whom are cisgender White British women who do not identify as having a disability. Author 2 has children, whereas Author 1 does not. Both interviewers had previous experience of conducting qualitative interviews and were trained in study-specific processes by the CI (Author 3) and qualitative lead (Author 7). Neither of the interviewers had an ARD, and they had no previous experience or expertise in this disease area. Interviewers maintained a broadly consistent approach to introducing themselves and the timeline before the interview, introducing themselves by email initially, and aiming to be friendly and informal in order to put women at ease.

**Figure 1.**
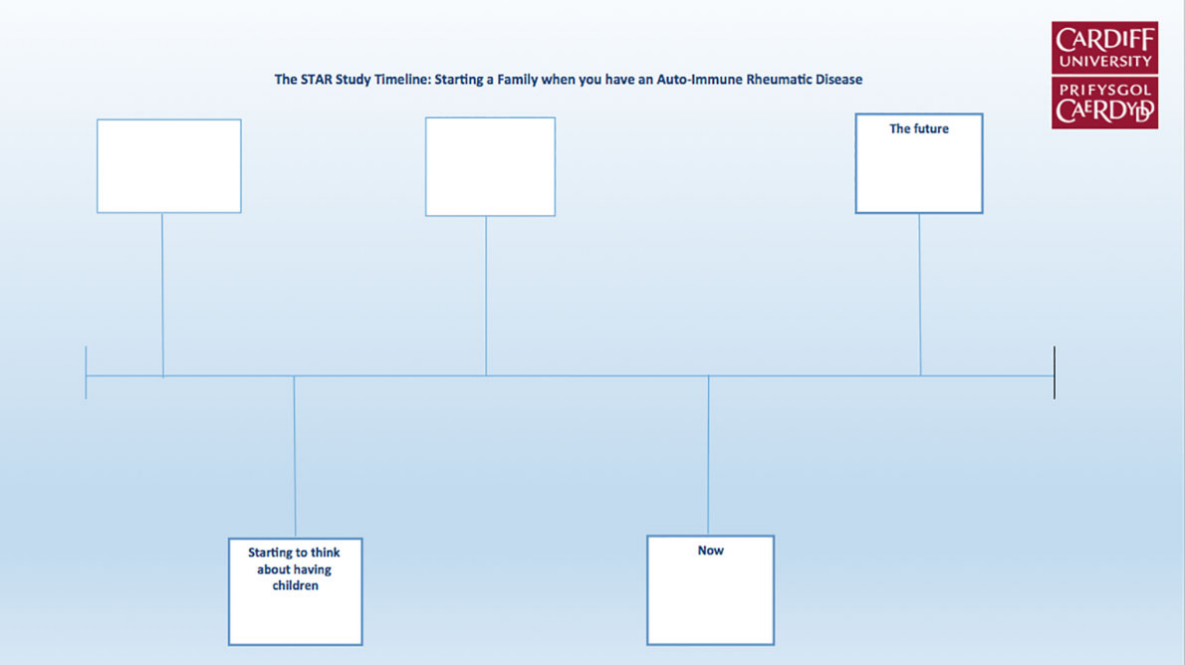
Timeline template.

Participants who agreed to an interview received a study resource pack approximately 1 week before the arranged interview date. The pack included stationary items consisting of paper, a timeline template, emoticon and colored stickers and colored pens, as well as an example key of colors to represent different feelings and physical symptoms, which they could choose to use or adapt. An example of the blank timeline template is provided in Figure 1. Alongside the stationary, participants were given colorful, easy-to-read documents that contained a list of topics in which we were interested, along with a set of instructions making it clear that participants could adapt the template, write as much, or as little as they wished, or even not write anything at all (see Supplementary File 1). We clearly stated that women did not have to talk about anything they did not want to. We highlighted that this exercise was to help them tell their own story, in whatever way they felt reflected their experiences.

The interviewers explained the study, their role, and the purpose of the timeline. Presenting the purpose of the timeline allowed the interviewers to highlight women’s ownership over the timeline elicitation tool and help to encourage the women to lead the interview and exercise more control over its direction. The process of outlining the timeline exercise and interview expectations is important for the purposes of obtaining informed consent (Groenwald & Bhana, 2015; Marshall, 2009)

### Ethical Approval and Informed Consent

Ethical approval for the study was granted by the Cardiff University School of Medicine Research Ethics Committee on October 20, 2016. All women provided written informed consent to participate in the interviews. The resource pack sent to women prior to interviews included a consent form for the participants to complete and return, with at least 48 hr provided to consider taking part, before interviews took place. Interviewers ensured that participants had an opportunity to ask questions and verified consent before the interview commenced to ensure that participants were happy to take part and to be audio-recorded.

### Data Collection and Processing

We completed 22 interviews with women in the STAR Family Study; 16 of these were carried out over the telephone and 6 face-to-face. All interviews were audio-recorded and were transcribed verbatim by a professional transcription company. Face-to-face interviews took place in the women’s homes. Telephone interviews took place over loudspeaker in a private space, enabling researchers to audio-record the interviews while maintaining confidentiality. Interviewers took field notes reflecting on the interview and the timeline elicitation approach. They wrote these up as soon as possible after the interview took place to ensure accuracy of the notes and to contextualize the interview.

### Data Analysis

We analyzed data from three sources: interviewer field notes, qualitative interview transcripts, and audio recordings of the interviews, which needed a flexible approach suited to methodological analysis incorporating data from multiple sources. We adopted Braun and Clarke’s (2006) approach to thematic analysis, a highly adaptable analysis that can highlight comparisons and encourage insight into unanticipated themes (Braun & Clarke, 2006; King, 2004). Field notes and interview transcripts were imported into NVivo V11 to facilitate analysis.

The first stage of analysis was familiarization, identifying areas of interest within different interactional concepts. Author 1 created an initial coding framework for both the telephone and face-to-face interviews, focusing on the methodological aspects of the data to provide context to the telephone interviews and to examine the interaction between the researcher, participant, and the timeline (when present). Author 1 searched for and identified themes within the telephone interview transcripts, using field notes and audio recordings to contextualize the information extracted. Discussions were held within the core qualitative research group to share reflections, review, refine, and name the themes identified (Authors 1, 2, 3, and 7) and reach agreement on the interpretation of the data. This approach has been deemed appropriate for qualitative research (Barbour, 2001).

## Findings

Table 1 presents participants’ demographic characteristics. The majority of participants had children, were employed and educated to degree level, and all participants identified as White. The overall mean length of the telephone interviews were 48 min and ranged from 20 min to 70 min in length.

**Table 1.** Demographic Characteristics of Interview Participants.

	Telephone Interviews *n* (%)
Family situation	
Thinking about having children	5 (31)
Pregnant	1 (12)
Have young children	9 (56)
Ethnicity	
British, English, Welsh, Scottish, or Irish	13 (81.3)
Other: non-European	2 (12.5)
Other: European	0 (0)
Employment	
Full-time employment	7 (37.5)
Part-time employment	6 (43)
Not employed or seeking work	1 (6)
Education: university degree or above	12 (82)
Age	Mean: 33 (*SD*: 4.8)
	Range 24–41

*Note*. *N* = 16.

Six themes relating to our research questions were identified in our methodology-focused thematic analysis: (1) use and adaptation of the timeline tool, (2) timeline exchange, (3) framing the interview: emphasizing that women are in control, (4) taking a lead, (5) jumping straight in, and (6) disclosing personal and sensitive experiences. The source of the data from which quotes are extracted and how the timeline was used (if present) in the corresponding interview are indicated at the end of each quote to provide context.

### Research Question 1: How Were the Visual Timelines Used by Women and Researchers in the Telephone Interviews?

Table 2 provides a summary of how interviewees used the visual timelines. Five of the sixteen women interviewed by telephone did not complete the timeline or an adaptation of the timeline, whereas in the face-to-face interviews, five of the six participants completed a version of the timeline.

**Table 2.** Participant Timeline Completion Details.

	Telephone Interviews *n* (%)
Copy of timeline returned to researcher	
Yes	0 (0)
No	11 (68.8)
Not applicable	5 (31.2)
Participant use of the timeline	
Timeline template	0 (0)
Bought medical notes to facilitate discussion	0 (0)
Created their own version of the timeline in note form	6 (37.5)
Created a version of the timeline using prompts and notes	2 (12.5)
Created a spider diagram version of the timeline	1 (6.3)
Participant used guidance notes to structure narrative	2 (12.5)
No timeline used	5 (31.3)

*Note*. *N* = 16.

#### Theme 1. Use and adaptation of the timeline tool

One of the benefits of using the visual timeline tool in the interviews was that it helped women to structure narratives and to talk about experiences that were important to them. In an instance where no timeline was used a participant said: “I’m sorry if this is all jumping around a bit, maybe I should’ve made notes” (telephone interview transcript and audio recording, no timeline used).

By contrast, another participant’s use of a timeline demonstrates how it prompted reflection and an opportunity to understand how she had structured her narrative: “I was just going to have another little look again, because I was having a look last night at the um, all the bits of paper again so err right” (telephone interview transcript and audio recording, timeline used).

Similarly, for the women who used a version of the timeline in the telephone interviews, it was observed by the researchers, and sometimes directly reported by participants, that making notes had helped to organize their thoughts chronologically. For example, one participant reported using the timeline guidance notes to frame her narrative, without making any written notes.

### Research Question 2: What Impact Did Using the Visual Timelines Have on the Generation of Data?

#### Theme 2. Timeline exchange

In the STAR Family Study, timelines were included primarily as an elicitation tool, and our data analysis focused on participants’ verbal narratives rather than on the timelines themselves. We did not ask for the timelines to be shared with the researcher before the telephone interviews, and we anticipated that women may add to or modify their timelines as they moved through their narratives. Therefore, the timeline was not visible to the interviewer. At the end of every telephone interview in which a visual artifact had been created, we did request a copy of it as a picture sent by telephone, scan sent by email, or paper version returned by post, as long as the women were happy to do so. Participants who had undertaken a telephone interview appeared to display some hesitancy about sharing their timeline:

I: I also wondered whether you wouldn’t mind, obviously I know that you, you probably want to keep your spider diagram, but if you wouldn’t mind taking a photo of it and sending it to me?

P: Yes if you would like to decipher my handwriting that’s absolutely fine (laughs)

I: Yeah that’s absolutely, yeah no that would be great if you wouldn’t mind sending a photo that would be fantastic. (telephone interview transcript and audio-recording, spider diagram version of timeline)

All the timelines were received by the research team for the face-to-face interviews, but none of the timelines produced by the telephone interviews participants were returned to the research team. By retaining ownership of their timelines during telephone interviews, women stayed in control of the data that they shared with the researchers, which was consistent with the ethos of the study. However, this meant that the visual data were not available to the researchers for analysis. In contrast, using the timeline as a tool during a face-to-face interview naturally includes a visualization of the timeline by the interviewer. The presence of the interviewer in person in the face-to-face interviews may have contributed to an expectation that participants would present their version of the timeline to them and might have influenced the content that they chose to include as a result.

The research team reflected that the timelines supported the primary analysis of the interview data, in those instances where they had been returned in the face-to-face interviews (reported in full elsewhere—Phillips, Pell et al., 2018). The timelines provided a structure around which to build a coding framework to organize the complex and varied content of narratives. The women’s experiences situated as a chronological journey within these timelines enabled us to identify key events and milestones. There was, therefore, a trade-off between empowerment of interviewees and the availability of visual data for analysis.

#### Theme 3. Framing the interview: Emphasizing that women are in control

The interviewers reiterated the purpose of the timeline and asked participants whether they had used the timeline at the beginning of interviews. This framing of the interview was emphasized in the telephone interviews, where the conversation was recorded from the outset:

Interviewer (I): I’d really love to hear about your experiences, what’s been important to you and to really let you lead on this so I’m happy to sit here and just listen

Participant (P): That’s kind of what I’m doing—doing a bit of a spider diagram of all factors that are important, or affect those, those decisions around that so it kind of really helped me think about things that might be useful to share (telephone interview transcript and audio recording, spider diagram version of timeline).

I: This interview to be honest with you, it’s not a typical interview I’m not gonna be firing questions at you in any way, shape or form. It’s really gonna be led by you and you telling us your story. You know treat me as a blank slate. Did you make any notes or did you do a little timeline at all to help tell your story. If you did you can use that, or you know you can start at any point you want to talk about really.

P: Yeah I think I’m just gonna start from the start. I think that’s the easiest way to do it. (telephone interview transcript and audio recording, notes version of timeline)

In both examples, the interviewer attempts to shift the interview power balance, continuing to set the scene of a women-centered approach by offering participants the opportunity to lead the interview and initiating a space for the women to talk freely. The framing of interviews from the outset by outlining the role of the participant as the narrator, with freedom to choose the topics, is an important element of empowering people to take control of their narratives (Veronesi, 2019).

The interviewer emphasized choice in the timeline activity when framing the interview:

I: did you manage to do anything with it ((the timeline))? Doesn’t matter if you didn’t, some women find it helpful to have written down some thoughts on what’s been important in their journey before we start, but it’s absolutely okay if you haven’t managed to do so. (telephone interview transcript and audio recording, notes version of timeline)

Indeed, some participants (*n* = 5) chose not to use the timeline in the telephone interviews. Most explained that this was because they were busy and didn’t have the time, although one woman highlighted the emotional challenge of putting her traumatic experiences in writing:

P: Sure, I had a look through everything that you sent to me, um and I chose not to write anything down.

I: Yeah.

P: Um I think, because I, I had a look at the sort of timeline part and um it just felt a bit difficult to put it on that really so.

I: Uh huh absolutely. Yeah it, it’s like I said, it’s just one of the tools that some women find useful, some women don’t so yeah it’s entirely up to you. (telephone interview transcript and audio-recording, no timeline used)

The timeline exercise was an optional activity for the women to use as a tool to make the interviews more woman-centered and to organize their thoughts. When participants had not completed a timeline, the interviewers used politeness strategies, communicating acceptance, emphasizing freedom of choice using warm and friendly tones to prevent participants’ feelings of guilt or shame before and after the interview process, for not completing the timeline activity, while still orienting the interview and the respective roles of the participant and interviewer.

#### Theme 4. Jumping straight in

The timelines were important in enabling women to focus their interview on the topic at hand and enabled them to move swiftly into their narratives. Once the interviewer had framed the interview and taken the participant through the mandatory ethical statements, the participant then progressed naturally into telling her story, as shown by the excerpt below. This suggests comfort in the situation, rapport with the interviewer, and a general desire to share experiences, which might have been encouraged by reflecting on experiences:

Okay so, I got married in 2013 and I was taking Leflunomide I think at the latter of at the end of 2012, I’d been on Leflunomide for oh a number of years like probably maybe 10 years, an awful lot of years and I noticed that my RA started to decline at that point and then so at the end of 2013 my RA was really quite bad. (telephone interview transcript and audio recording, notes version of timeline)

The timeline exercise helped participants reflect on and frame their journey before telephone and face-to-face interviews, which may have reduced preinterview anxiety about “saying the wrong thing,” which has been reported in narrative interview studies (Holt, 2010).

#### Theme 5. Taking a lead

The timelines encouraged discussion around things that might not have been prompted by the interviewer but that the participant clearly felt were important to discuss as part of their journey, demonstrating authoritative behavior as a result of using the timeline. There were times in the telephone interviews where the timeline became a physical prompt, so the participant could check whether they had covered everything they wanted to: “So yeah that’s something and there was one other thing I was going to say about after the baby was born but I can’t remember what it is now I’m trying to scan my notes ((laughs)).” Once the participant found the part in her notes she was referring to, she then continued with her narrative:

yeah I’ve known about how my condition might affect the baby so just a bit of nervousness about the <<condition>> a little bit of that and if there are any congenital heart defects…I kind’ve feel like there’s a couple more things because of my condition, those couple more things are all very serious things.” (telephone interview transcript and audio recording, timeline in note form)

The field notes indicated that at the end of the interview, one woman who took part in a telephone interview requested for the recorder to be switched back on and for the interview to be continued, so that she could raise something that she had written in her notes but not yet spoken about:

At the end of the interview the participant checked the notes that she had made and realized that she had not covered one topic that she had previously written down. She therefore requested permission for the recorder to be switched back on and for the interview to be continued. (extract from researcher field notes and audio recording, telephone interview)

There were occasions when participants sought direction from the interviewer: “So that’s sort of, I don’t know if you’ve got any questions around that, that would be helpful to explore for you?” (telephone interview transcript and audio recording, timeline in note form). Here, the participant assessed the relevance of their narrative in response to the interviewer’s research goals, thereby orienting to a new frame, which sought the interviewer’s confirmation and response. This indicates a subtle resistance to the original framing of the interview, emphasizing women’s freedom to share their stories in their own way, as there was still a desire to provide information that was of interest to the researcher.

### Research Question 3: What Impact Did Visual Timelines Have on the Quality of Data Produced in Telephone Interviews?

#### Theme 6. Disclosing personal and sensitive experiences

Telephone interviews elicited personal and sensitive accounts of the women’s experiences. The timeline, coupled with our woman-centered study ethos, encouraged this by giving the women freedom to navigate the interviews and tell their own stories. The process of holding preinterview conversations regarding the timeline could have helped in initiating the development of a relationship and establishing trust prior to the telephone interview. Interviewers emphasized choice, freedom, and interest in the women’s stories, as well as highlighting choice in their interview setting, date, and time. The rich quality of narratives elicited in the telephone interviews in terms of the length of interviews, level of detail, and range of topics covered was comparable with the face-to-face interviews carried out within the same study. This highlighted the importance of developing a rapport before the interview. Developing and building rapport with participants is a continuous process, which needs to be revisited and nurtured to establish trust when it comes to the facilitating the interview.

Using the timelines helped capture the meaning attached to important events in the participant’s life (Leung, 2010) and illustrated the participant’s lived experiences of ARD. The telephone interviews within this study produced detailed and open accounts, with personal and sensitive disclosures being made. Participants’ innermost emotional and physical struggles were discussed:

(I had to take time off work) every 3 or 4 months because the RA wasn’t, it wasn’t completely stable, but it wasn’t as stable as it could’ve been and that year was a huge, huge struggle. I struggled to stay in work I had a lot of time off. I struggled emotionally with it and me and (my husband) physically struggled to kind of, want for a better phrase, to have enough sex to make a baby because I was in so much pain and so exhausted all of the time. (telephone interview transcript and audio recording, no timeline used)

## Discussion

In this article, we reflect on how visual timelines were used in telephone interviews as part of the STAR Family Study, what impact they had on the generation of data, and the quality of data generated using this method. The visual timeline method enabled women to lead the interviews and elicited long, rich narratives and personal, sensitive accounts of their experiences. Interviewer characteristics and study ethos were important in determining how the timelines were introduced and used. Consideration of how these contextual factors interact with the use of timelines is important when selecting appropriate methods for qualitative studies.

Use of the timeline elicitation encouraged women to take control over the direction of the interview, ownership of stories, and disclosure of rich, personal accounts. The interviewer’s position of dominance and control within a standard semi-structured interview is well-established (Gillham, 2005). Timelines can be used as a tool to reduce this power imbalance, particularly among vulnerable or marginalized groups (e.g., Berends, 2011; Goodrum & Keys, 2007). In this study, the woman-centered ethos that aligned with feminist research principles (Brooks & Hesse-Biber, 2007; Hesse-Biber, 2011; Landman, 2006; Tong & Fernandes Botts, 2017), along with freedom to adapt the timeline or not use it at all, ultimately set the scene for participants to play a role in leading the interview from the beginning. The timeline tool can be used to reduce some forms of power imbalance (Vogl, 2013). However, it is difficult to remove this altogether (Packard, 2008), particularly with marginalized groups (Mannay, 2016), and there were occasions where participants sought direction from the interviewer.

In addition to the tools used within interviews, interviewer characteristics and the approach used can influence power relations in interviews (e.g., Coffey, 1999; Johnson, 2018; Lomax, 2015; Mannay, 2016; Rose, 2007). The data produced in this study’s telephone interviews elicited rich, long narratives where the women offered personal and sensitive accounts of their experiences (Phillips, Pell et al., 2018). While there is uncertainty and ambiguity in the literature over telephone interviewing and its ability to elicit participant openness (Novick, 2008), our study supports the growing notion that telephone interviews can be just as powerful as face-to-face interviews in their ability to collect rich, in-depth data (e.g., Lee et al., 2013; Stephens, 2007; Trier-Bieniek, 2012). Although it can be more difficult to establish a good rapport in telephone interviews than face-to-face (e.g., Hermanowicz, 2002; Shuy, 2003), the timelines may help mitigate this by giving participants ownership over their narratives, an opportunity to reflect and organize their thoughts prior to interview, and enable them to move rapidly into discussing sensitive and emotive issues, creating a level of rapport that may still be comparable (Deakin &Wakefield, 2013).

There have been debates previously around the coproductive nature of the timeline method and its integral part of the interview (Gauntlett, 2007). These include shifts in power imbalance, questions of anonymity and confidentiality (Berends, 2011; Marshall, 2017), as well as raising the possibility of participants’ feelings of oversharing (Adriansen, 2012). In this study, we chose to use the timeline as a preinterview tool, focusing our analysis on the verbal data provided by participants rather than on the timeline itself. This enabled women to complete the timeline autonomously, ahead of the interview, allowing time for them to decide what not to share rather than “coproducing” the timeline with the researcher.

An unanticipated effect of women completing timelines autonomously prior to telephone interviews was that participants chose to retain ownership of the timelines after the interviews rather than giving them to the researchers to use during analysis. In keeping with our study ethos and woman-centered approach, we felt it was important for the women to maintain ownership and control of their personal stories and thus their timeline to give them a platform to organize and frame their narratives before the interview and thus facilitate and elicit in depth narratives. This could have had an impact on the researcher–researched relationship due to the researchers’ potential dependence on the participants’ willingness to share the timeline, which again would have shifted power dynamics within the relationship (Råheim et al., 2016).

Disadvantages of the approach that we used in this study are that visual data from the timelines could not be used to query and prompt during telephone interviews, and visual data were lost as timelines were not made available to researchers to use during analysis. The research team reflected that timeline templates provided to them following the face-to-face interviews completed as part of the STAR Family Study were useful in supporting data analysis (Phillips, Pell et al., 2018), enabling us to look at how the participants had organized their narratives chronologically and words or images that were emphasized. This encouraged the interpretation of complex, layered data in the visual timelines, which may not have been easily conveyed through narrative. Understanding and balancing these issues is important in considering how timelines are used, and depending on the focus of a particular study, it may be useful to request a copy of the timeline in advance of interviews.

### Reflection and Future Research

Limitations of this study include a relatively small sample size of women, over half of whom were highly educated to degree level and there was no ethnic diversity. There was a strong element of self-selection in our study, and we are unable to tell how willing our participants were to share their stories compared with the wider population of women of childbearing age who have an ARD.

Interviewer positionality is well-established as affecting all research, particularly in vulnerable populations (Mannay & Creaghan, 2016; Rose, 2007). Most women had not received adequate support or felt listened to by health professionals in their prior experiences (Phillips, Pell et al., 2018). The provision of a safe space for them to share their lived experiences and feelings surrounding this might have been a rare opportunity and consequently encouraged honesty and richness in their accounts due to the cathartic experience within this. Women’s possession of their own timeline may have validated the ownership of their stories and thereby the freedom to share what they felt comfortable in sharing, perhaps reducing the ethical dilemmas raised in previous studies (Adriansen, 2012; Berends, 2011; Lomax, 2015). Author 1 and Author 2’s roles as White British women, and Author 2’s experience as a mother might have encouraged rapport due to relatability. This coupled with our genuine interest in the women’s journeys appeared to have inspired the women to be open with the interviewer, which might not have been the case if the interviewer were a different person. This helped them to gain insight into their own experiences and therefore reflect on and process their painful experiences in a healthy way, sometimes giving them a reported sense of closure (Gabb & Fink, 2015). This reciprocity and interactivity again helps to reduce patient discomfort and can reduce power imbalance (Eder & Fingerson, 2002; Rose, 2007).

## Conclusion

Timelines provide the opportunity for flexibility and diversity in the formation of narratives—a way of straying outside the boundaries and thinking creatively—which can be adapted for specific studies. In our study, we demonstrated that there using timeline-facilitated interviews in telephone-based qualitative research is feasible and has value in encouraging a woman-centered approach, enabling women to reflect on and organize their experiences and giving them control over their narratives. There was considerable depth and insight in the narratives received from our participants in the telephone interviews. There were specific aspects of the data generation process that were altered in the context to telephone (as opposed to face-to-face) interviews with regard to the researcher–participant relationship and ownership of the timelines that need to be considered when using this approach. The use of timelines as elicitation tools within telephone interview studies should be further explored with a wider range of populations to examine the value of these tools and methods in obtaining high-quality data during telephone interviews. We also suggest further research to explore the utility of other visual and creative methods in telephone (and other remote) methods of interviewing.

## Supplemental Material

Supplementary_File_1 - Using Visual Timelines in Telephone Interviews: Reflections and Lessons Learned From the Star Family Study Click here for additional data file. Supplementary_File_1 for Using Visual Timelines in Telephone Interviews: Reflections and Lessons Learned From the Star Family Study by Bethan Pell, Denitza Williams, Rhiannon Phillips, Julia Sanders, Adrian Edwards, Ernest Choy and Aimee Grant in International Journal of Qualitative Methods
